# Levels of total serum IgE and specific IgE sensitization profile in patients with atopic dermatitis

**DOI:** 10.1186/1939-4551-8-S1-A142

**Published:** 2015-04-08

**Authors:** Rafael Teixeira Figueredo Poleshuck, Tatiane Cristina Marques, Norma Rubini, Fernanda De Lima Barros Limongi, Liciene Neves Portela, Fernando Samuel Sion, Carlos Alberto Morais De Sa

**Affiliations:** 1 Federal University of the State of Rio De Janeiro, Brazil

## Background

Atopic dermatitis is a multifactorial disease associated with elevated production of IgE and sensitization to multiple food and inhalant allergens.The aim of this study was to assess the levels of total serum IgE (IgE) and specific IgE (sp IgE ) for Dermatophagoides pteronyssinus (Dp), Blomia tropicalis (Bt), egg white, casein, alpha-lactalbumin and beta-lactoglobulin in patients with atopic dermatitis (AD); and the influence of gender, age and severity of disease in these parameters.

## Methods

We conducted a retrospective, cross-sectional study with patients with AD, between 2-18 years old and who had regular follow-up. Data were collected from medical records and the LAPIA Database. IgE and sp IgE measurements were performed by ImmunoCAP.

## Results

We analyzed 30 patients, 50% (15) males, mean age = 10.3 years (SD = 10.6). IgE levels were elevated in 79% of the patients, 55% had sp IgE sensitization to mites; 36% to egg white; 34.6% to alpha-lactalbumin; 26.9% to beta-lactoglobulin and 30.76% to casein. In children, the percentage of sensitization to mites was 35.29% and 76.9% in teenagers (p = 0.02). The percentage of sensitization to mites, egg, casein, alpha-lactalbumin and beta-lactoglobulin in patients with mild AD was, respectively, 25%; 11.1%; 12.5%; 11.1% and 14.3%; in those with moderate / severe AD was: Dp and Bt (75%), egg white (88.9%), casein (87.5%), alpha-lactalbumin (88.9%) and beta-lactoglobulin (85.7%) [p <0.05].

## Conclusions

We observed a high percentage of patients with elevated IgE levels, a large proportion with sp IgE sensitization to mites and a third with sensitization to food. Our data indicate that sensitization to mites is more common in adolescents and that patients with moderate / severe AD have a higher risk of sp IgE sensitization to mites, egg and milk proteins.

